# Newborn Cry-Based Diagnostic System to Distinguish between Sepsis and Respiratory Distress Syndrome Using Combined Acoustic Features

**DOI:** 10.3390/diagnostics12112802

**Published:** 2022-11-15

**Authors:** Zahra Khalilzad, Ahmad Hasasneh, Chakib Tadj

**Affiliations:** 1 Department of Electrical Engineering, École de Technologie Supérieur, Université du Québec, Montréal, QC H3C 1K3, Canada; 2 Department of Natural, Engineering and Technology Sciences, Arab American University, Ramallah P.O. Box 240, Palestine

**Keywords:** cepstral features, sepsis, RDS, SVM, MLP

## Abstract

Crying is the only means of communication for a newborn baby with its surrounding environment, but it also provides significant information about the newborn’s health, emotions, and needs. The cries of newborn babies have long been known as a biomarker for the diagnosis of pathologies. However, to the best of our knowledge, exploring the discrimination of two pathology groups by means of cry signals is unprecedented. Therefore, this study aimed to identify septic newborns with Neonatal Respiratory Distress Syndrome (RDS) by employing the Machine Learning (ML) methods of Multilayer Perceptron (MLP) and Support Vector Machine (SVM). Furthermore, the cry signal was analyzed from the following two different perspectives: 1) the musical perspective by studying the spectral feature set of Harmonic Ratio (HR), and 2) the speech processing perspective using the short-term feature set of Gammatone Frequency Cepstral Coefficients (GFCCs). In order to assess the role of employing features from both short-term and spectral modalities in distinguishing the two pathology groups, they were fused in one feature set named the combined features. The hyperparameters (HPs) of the implemented ML approaches were fine-tuned to fit each experiment. Finally, by normalizing and fusing the features originating from the two modalities, the overall performance of the proposed design was improved across all evaluation measures, achieving accuracies of 92.49% and 95.3% by the MLP and SVM classifiers, respectively. The MLP classifier was outperformed in terms of all evaluation measures presented in this study, except for the Area Under Curve of Receiver Operator Characteristics (AUC-ROC), which signifies the ability of the proposed design in class separation. The achieved results highlighted the role of combining features from different levels and modalities for a more powerful analysis of the cry signals, as well as including a neural network (NN)-based classifier. Consequently, attaining a 95.3% accuracy for the separation of two entangled pathology groups of RDS and sepsis elucidated the promising potential for further studies with larger datasets and more pathology groups.

## 1. Introduction

According to the World Health Organization (WHO), millions of children die every year globally. It was also indicated that the majority of deaths among children occur under the age of one month; for example, in 2020, 2.4 million children died globally in the first month of their lives, adding up to 47% of all child deaths being under-five mortality, which was 40% in 1990 [1]. This shows that the neonatal mortality rate is increasing globally. The WHO also presented the main pathological causes that may lead to neonatal death, where 75% of neonatal deaths usually occurred during the first week of life. Some of these pathological causes included Neonatal Respiratory Distress Syndrome (RDS) and sepsis.

The reason behind the RDS is unknown; however, it is often associated with surfactant deficiencies [2]. From 2016 to 2020, RDS was among Canada’s leading causes of post-partum mortality, and nearly 100 newborns lost their lives due to this pathology during the mentioned years [3]. Typically, the clinical diagnosis of RDS is carried out via a series of tests which include recording echocardiography, collecting blood samples, measuring the oxygen levels in the bloodstream through pulse oximetry, and chest and lung radiography [4]. RDS is, thus, identified by breathing difficulty in a newborn and red or blue color of the face and lips and should be diagnosed at an early stage since it could lead to many developmental difficulties such as vision or hearing impairment, learning challenges, and mobility problems. However, it is worth mentioning that there is no determined test for diagnosing RDS or ruling out the possibility. 

On the other hand, sepsis was among the top 10 pathological causes that led to the mortality of infants in Canada between 2016 and 2020; it took the lives of more than 185 newborns [3]. In a general sense, sepsis is an infection that entails the blood and it may lead to, or be associated with, several other pathological conditions such as hypothermia, hypotension, or even RDS [3,5]. Neonatal sepsis is clinically diagnosed based on having at least two of the following symptoms: high or low heart rates, feeding problems, lethargy, fever, hypotonia, convulsion, hemodynamic abnormalities, and apnoea that lasts for more than 20 s [6]. Therefore, the present clinical tests for diagnosing sepsis take time and have a moderate risk of producing false negative and false positive results. Consequently, it is of great significance to promptly identify this pathology in the newborn to start the treatment procedure before the onset of symptoms. 

It can thus be seen that both pathologies require intrusive and in-depth clinical tests to be diagnosed accurately, and they are associated with high mortality and morbidity rates for newborns. Furthermore, it has been shown that sepsis and RDS are closely associated and entangled [5], and sepsis is one of the main causes of RDS [6]. Therefore, studying and analyzing these two infant pathologies by the means of a simple, automated, and non-invasive tool, such as a newborn cry-based diagnostic system (NCDS), is preeminent and essential. This system can serve as a tool for early recognition and accurate diagnosis of these infants’ pathologies, which greatly contributes to acquiring the necessary treatment for the infant before the onset of symptoms and, thus, preserving the infant’s life. In addition to that, the distinction between these two pathologic groups (sepsis versus RDS) will be lucrative in demonstrating that the concept of distinguishing neonates with certain pathologies from other pathological infants is an auspicious goal. 

Typically, infants communicate with those around them through crying; it is a combination of vocalization, coughing, choking, and interruption, which includes a diversity of prosodic and acoustic features at different levels [7]. Recently, the analysis and understanding of infant crying signals have been receiving growing attention from researchers and data scientists, with the aim of diagnosing the infant’s pathology in its early stages. In this respect, it has been shown that infant cries provide important acoustic parameters or characteristics that should be taken into consideration, studied, and analyzed while monitoring the first days of an infant’s life [7,8]. Furthermore, the cry signals of unhealthy infants usually contain unique features or characteristics that differ from healthy ones [7]. Consequently, pathological cry signal analysis and classification can be used as a valuable tool for predicting and recognizing neonatal diseases before the onset of the symptoms. 

By using the cry signals, various audio feature categories can be computed and generated, including cepstral, prosodic, and spectrograms, that have been widely used and applied to different research related to music, speech, and environmental sounds. These categories have separately been used for the identification of pathologies in newborns, and few attempts studied the combination of these features for the same purpose. In this research work, we aim to combine two feature categories, namely the cepstral domain and the prosodic domain, and then employ the combined features for training the classifiers. The ultimate goal of this research is thus to investigate the capacity of machine learning methods to discriminate between the septic and RDS cries, by using the combined feature set of the prosodic and cepstral domains. The characterization of different pathological patterns using the audio features would enable the development of an early and accurate diagnostic system that aggregates various audio feature categories to assist the early identification of abnormal acoustic behavior and link it to the early signs of a specific infant pathology. To the best of our knowledge, the question of utilizing different audio domains with a hyper-tuned machine learning model to classify infant RDS cries from infant septic cries has not been considered yet. 

The presented study was proposed to address three main challenges in the field of pathological cry analysis. Firstly, despite the wide range of valuable research proving that the newborns diagnosed with a pathology cry differently than healthy newborns, there is no study where the cry signals of two pathology groups are compared to the best of authors’ knowledge. Secondly, there is an inadequate number of studies that target sepsis and RDS; more specifically, the studies that target cries associated with RDS as a single pathology group (as opposed to being a part of an entire “pathologic” group) are scarce and the few existing studies never obtained an accuracy of more than 75%. Third, low-income countries suffer the most from infant mortality rates, which is due to their lack of adequate monitoring equipment, low number of pediatricians, and lack of resources. Child mortality risks in low-income countries are 16 times higher than high-income countries [9], which calls for designing non-complex, fast and efficient tools for early diagnosis.

This study is the first to answer the question of pathologic versus pathologic that aimed to take the existing methods and algorithms and design a simplistic, yet efficient system, that requires only the everyday commercial tools. Our design benefits from a unique dataset owing to multiple factors. Firstly, no well-defined procedure or specific conditions were imposed during data collection phase; the data were collected in maternity rooms, Neonatal Intensive Care Units (NICUs), etc., where noise of medical equipment and staff and newborn’s guardians’ chatter was also present. Second, the recording was carried out by a simple handheld recorder, which can be found even in deprived areas of the world where the newborn mortality rates are at its highest. Third of all, data collection does not necessitate even as much as simply touching the newborn which makes our design a truly non-invasive method. 

Despite the ever-growing use of computationally expensive tools, and also the perspective where crying is thought of as a pre-speech signal, we employed conventional tools from different fields such as musical applications, non-speech audio analysis and processing. We fused and optimized them so that the final design remains simplistic yet achieves the compatible performance of the state-of-the-art methods. The combination of the prosodic domain and cepstral domain features, which could lead to a new feature set that takes advantage of each domain and thus improves the linear separation between the two pathologies, is considered here by combining GFCCs and HR feature sets for the first time in the study of diagnostic analysis of the cry signal.

The rest of the paper is organized as follows. The related work on infant pathologies classification techniques is discussed in Section 2, while Section 3 describes the proposed methodology, including a description of the dataset and participants, features extraction, and modeling, followed by a description of the different machine learning methods that have been tuned and applied to this classification problem. Section 4 presents and discusses the obtained results. Finally, Section 5 presents conclusions and outlines future work.

## 2. Related Work

In the early years of pathological infant cry signal analysis and classification, numerous artificial intelligence (AI) and machine learning (ML) techniques were proposed and developed. Researchers can find many research works on infant pathological cry analysis and classification in [7,10]. One can see that researchers continue to apply new machine learning methods to classify infant cry signals into normal and pathological records; for example, see the recent works in [11,12]. However, some of the current research works include identifying pathologies such as hypo-acoustic [13], asphyxia [14,15,16], hypothyroidism [17], septic [18,19], RDS [20], and autism spectrum disorder (ASD) [21]; additionally the authors in [8,22,23,24] have investigated different infant pathologies. In particular, the asphyxiated infant crying signals have been identified using different ML methods, including a deep feedforward neural network (DFNN) model [14], a support vector machine (SVM) model [15], and a convolutional neural network (CNN) approach [16], and achieved accuracy rates of 96.74%, 98.5%, and 92.8%, respectively. In addition, hypothyroidism has been studied in [17] using a Multilayer Perceptron (MLP) classifier, and achieved a classification accuracy of 88.12%. Two groups of authors investigated sepsis in newborns recently; the authors in [18,19] have developed a machine learning-based CDS for identifying septic newborns and reached an accuracy of 83.9% using majority voting, while the authors in [19] attained 89.99% using entropy-based features. Furthermore, ASD in [21] and RDS in [20] have been identified based on a SVM and reached accuracies of 96% and 73.8%, respectively.

Normal and hypo-acoustic infant cry signal classification has also been proposed in [13] using general regression Neural Networks (NNs) and reached 99% accuracy. Therefore, most of the existing NCDS models have mainly focused on investigating one pathology individually versus healthy cases. The authors in [8,11,22,23,24] have proposed to classify different pathological types of infant cry signals, namely: normal, deaf, asphyxia, hungry, pain, jaundice, and premature from the healthy group. Moreover, their proposed model is based on a combination of wavelet packet-based features and an Improved Binary Dragonfly Optimization-based feature selection method, and they conducted several classification experiments of two-class and multi-class of crying signals and achieved promising results. 

As mentioned before, different audio feature categories can be extracted from infant cry signals using the following domains: cepstral domain, prosodic domain, time domain, image domain, and wavelet domain [7]. Each domain represents different aspects of the infant’s cry signal and they each present specific information and characteristics. Compared to the time domain features, which are more sensitive to the background noise, the cepstral domain features have been shown to be more robust in modelling characteristics and covering variations within infant crying signals [7]. These frequency-domain features can be computed using different mathematical tools, including Mel-frequency cepstral coefficients (MFCCs), Linear Prediction Cepstral Coefficients (LPCCs), Bark Frequency Cepstral Coefficients (BFCCs), Gammatone Frequency Cepstral Coefficients (GFCCs), and Linear Frequency Cepstral Coefficients (LFCCs). Indeed, cepstral features have been widely used in the field of speech processing and recognition, and the most frequently used ones to identify infant pathologies are MFCCs, LPCCs, and LFCCs, which have shown better performance compared to time domain features. In particular, MFCCs are the most used and tested features to identify infant pathologies; for example, asphyxia in [15,16], and hypothyroidism in [17], and achieved promising accuracies as presented above. Liu et al. also used MFCCs along with LPCCs and BFCCs and based on a NNs model to identify infant cry reasons and the results showed that BFCCs produced the best classification rate of 76.5% [25]. Furthermore, the authors in [26,27], showed that LFCC performed better than MFCC in distinguishing high-frequency audio signals such as female voice and infant crying signals. On the other hand, GFCCs have been shown to be powerful descriptors in non-speech recognition tasks, such as emotion recognition [28,29], understanding the reason behind the crying of infants [30], and automatic speech recognition [31]. There is one recent study where authors employed Gammatone Cepstral Coefficients (GTCCs) that are based on the time-representation of the signal for identifying infants suffering from Hypoxic Ischemic Encephalopathy (HIE) based on their cry signal [32]. It is noteworthy to highlight that our study employs the frequency-representation by extracting GFCCs since they have proved successful in audio recognition tasks [33].

Prosodic domain features, which include high-level information such as formants, intensity, duration, harmonicity, and unvoiced regions, also contribute in improving the discriminative ability between the crying signals and thus identifying the type of the infant cry signal; an example of this is the identification of asphyxia in [14]. It has been shown that attaching these features together with frequency domain features contributes to extracting both physiological and physical information from acoustic signals [7]. Furthermore, image domain features, such as the spectrogram which is a time-frequency image representation of an audio signal and includes both acoustic and prosodic information, can be used to distinguish between healthy and unhealthy infant cries. It has been widely shown that feeding spectrograms into machine learning algorithms also plays an important role in enhancing the classification of different infant crying signals [34,35,36,37]. It is, therefore, obvious that each domain contributes to the classification of infant crying signals, and thus the mechanism of generating a combined feature set that takes advantage of different domains deserves to be considered and investigated.

Several relevant recent research works have already shown promising enhancement with combined features to the problem of infant cry signals analysis [14,37,38,39]. More specifically, Ji et al. showed that combining MFCC features with weighted prosodic features contributed in improving the classification rates of the asphyxiated infant cry signals using a deep learning approach [14]. In addition, a combined NNs model that combines summative and temporal features was proposed for infant cry classification and outperformed the independently-trained temporal and summative networks [38]. In addition to that, the authors in [37] have shown that using hybrid features of the prosodic, spectrogram, and waveform classified by a CNN model produces better infant sound classification rates for the two different datasets. Moreover, a more recent study has investigated the use of hybrid features of MFCC, Spectral Contrast, Chromagram, Mel-scaled Spectrogram, and Tonnetz based on CNN and DFNN learning models [39]. The results have shown that deep learning models performed better with hybrid features compared to the use of single feature of MFCC. It was shown that combining DCNN with RBF-SVM was capable of achieving up to 88.89% accuracy in classifying infant cries based on the reason of crying [40]. Incorporating deep learning networks and combining them has shown the potential for state-of-the-art performance. For example, Khatun et al. [41] proposed a DCNN-LSTM classifier with self-attention model, which was capable of attaining an accuracy of 99.93% for human activity recognition purposes. In another study for classifying MRI brain tumor, authors implemented CNN with PCA in the feature extraction step and fed these features to different machine learning classification algorithms, which yielded a remarkable 99.76% accuracy [42].

To summarize, most of the existing models focus on analyzing infant cry signals to identify one pathology by using different machine learning techniques. To the best of our knowledge, no studies have addressed classifying RDS cries from sepsis cries using machine learning methods. Moreover, we noticed a lack of studies that give attention to the question of combining cepstral domain features and prosodic domain features to be used in classifying different infant pathologies. Therefore, finding the optimal combination of cepstral and prosodic domains, followed by a fine-tuned machine learning algorithm, remains an open question and needs further research investigations. Therefore, this paper proposes to use different machine learning techniques that use a combined feature set of cepstral and prosodic. The main contributions of this research work can, thus, be summarized as follows:

- Different machine learning techniques were used to classify RDS cries from sepsis cries. In this regard, all used ML techniques were fine-tuned to give the best classification rates. Our fundamental goal is to prove the concept that a NCDS can be built, starting with these two pathologies that are most common in newborns.
- It is the first demonstration that GFCC features, and HR descriptors can be combined and used to support the diagnosis of pathologies in newborns. In this regard, we show that combining the two feature sets played an important role in improving the classification results.
- An accuracy of 95.3% with 0.95, 0.95, and 0.95 precision, recall, and F-score, respectively, were obtained using a fine-tuned SVM to distinguish between RDS and sepsis cries.

## 3. Materials and Methods

**Figure 1:**
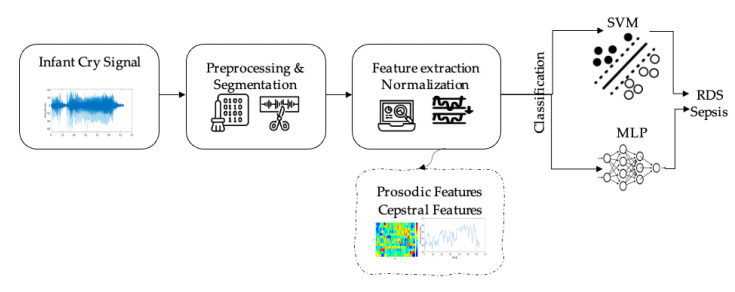
The workflow of the proposed model for different infant pathological classification and using the crying signals.

It is well known that extracting the most significant efficient features from given data plays an important role in simplifying subsequent tasks, such as the classification process, and thus leads to more accurate results. In this proposed work, we propose a combined feature set specifically for the classification of infant pathological cries. As shown in Figure 1, the workflow of the proposed model involves four main stages, which can be summarized as follows: (1) signal preprocessing and segmentation, (2) features extraction, selection, and modelling (3) machine learning model, and, finally, (4) pathological cry classification. 

### 3.1. Dataset Description

The samples included in this study were acquired as a result of collaboration between Saint-Justine children’s hospital in Montreal, Canada and the Al-Raee and Al-Sahel hospitals in Lebanon. As explained in our previous works [24], the cries in our dataset were collected from the newborns regardless of their race, gender, weight, or cry stimulus (pain, hunger, etc.). These cries have been collected with a common digital 2-channel Olympus handheld recorder with a 16-bit resolution and 44,100 Hz sampling frequency placed in the 10-to-30-cm vicinity of the newborn’s mouth. The cries were recorded in the hospital environment including maternity rooms and NICUs with no well-defined procedure and in the presence of noise. The health status of the newborn was determined based on several screening tests performed after birth and the cry signals were labeled as healthy or with the diagnosed pathology group based on medical reports accordingly. The gestational age, race, reason of crying, babies age, weight, and APGAR score were all noted. These considerations make our dataset a real and comprehensive one that can study newborns and propose a real-world solution in designing newborn cry diagnostic systems. The age of the babies in this study ranged from 1 to 53 days old, since it is not until the end of the second month of life (53 days to be precise) when newborns gain control of the vocalizations they produce [43]. Prior to this age, any vocalization is controlled by independent biological rhythms and thus it could be an indicator of newborn’s health. Moreover, the restructuring of the supralaryngeal vocal tract takes place around 3 months of age [43]. Therefore, this study excluded the newborns with a postnatal age more than 53 days.

It is well known that a majority of pathological studies encounter the same main obstacle, which is data acquisition. This challenge is attributable to several factors: (1) the unpredictability of whether a newborn with the targeted pathology groups will be observed during the data collection period, (2) acquiring the ethical and technical approvals to incorporate a cry sample in the database is a timely and difficult process which may result in losing some of the samples and (3) obtaining the newborns’ guardians’ consent to record their newborn’s cry and then add it to the database is quite challenging.

Given all these obstacles, we tried to segment each recording to multiple expiration segments in order to overcome the data limitation challenge and better study the characteristics of pathological newborn cries. There was a total of 53 recordings from 17 newborns for sepsis, and 102 recording from 33 newborns for the RDS pathology groups. These recordings had an average of 90 s including silence, hiccups, inspiration cries, expiration cries, and background noise. The original newborn cries were recorded with different durations ranging from 1 to 4 min with an average of 90 s, obtaining up to 5 recordings per newborn, which was inadequate for classification purposes. As explained in data preprocessing section, multiple EXP segments were extracted from each recording, which were later treated as an individual sample; these formed the 2264 samples mentioned in Table 1 with an average length of 0.71 s for sepsis and 0.74 s for RDS.

In this study, the expiratory cries of newborns diagnosed with sepsis and RDS were included with 17 and 33 newborns in each group, respectively. In order to have a well-balanced and homogenous study, we selected the same number of samples from each pathology group, Table 1.

Finally, it is noteworthy to mention that despite the fact that RDS is mainly attributable to prematurity, term newborns are often misdiagnosed or not considered for RDS. Although the occurrence of RDS in term newborns is exiguous compared to the preterm newborns, several studies show that a notable number of term-born neonatal hospital admissions are still due to RDS every year [44,45], accounting to a total of around 8%. Another study showed that 43% of term-born respiratory failures are due to RDS, which is a serious alert to not rule out RDS in term neonates [46].

### 3.2. Data Preprocessing

The cries of infants in our dataset have been processed by our previous colleagues in order to remove silence, filter, and segment each recording. Each recording was segmented and assigned with multiple labels. For example, the expiratory cries were marked as EXP, or the phonation during inspiration was labeled INSV, which represents a voiced inspiratory cry segment. These labels were attached by the means of WaveSurfer software. In the present study, we used the EXP segments of each cry recording and treated each segment as a sample. As has already been stated, one of the main challenges in any biomedical research is the limitation of data, especially in a problem such as this study, where the chances of observing a newborn suffering from a certain pathology are not predictable. Therefore, by segmenting each cry signal, we solved this challenge to a fair extent. 

### 3.3. Features Extraction and Modelling

As mentioned before, the main focus of this study is the extraction and the study of feature sets that are capable of representing the differences in newborn cries associated with two entangled groups of pathologies, RDS and sepsis. The cry signal is non-stationary and dynamic, which calls for the study of both short-term and spectral features. Furthermore, it has been shown that although MFCCs are the most commonly used features owing to their high performance [20], GFCCs outperform them in terms of less computational costs and better performance [47]. Thus, we studied GFCC features as short-term representations of the cry signal, as well as the harmonic factors that capture the spectral behaviour of resonance frequencies in newborn cries. We studied these features individually and then fused them to test the performance of the NCDS, considering both short-term and spectral features. The following sections expound on the procedure needed for the acquisition of these features.

*Gammatone Frequency Cepstral Coefficients (GFCCs)* are considered an alteration of the MFCC feature inspired by the biological model of the auditory system. GFCCs employ the equivalent rectangular bandwidth (ERB) bands instead of triangular bands and mimic the cochlear spectral structure in mapping the frequencies [47]. The spectrogram representation of Gammatone-Frequency is called cochleagram. A cochleagram is expected to have fair performance with pathologic newborn cry signals since the lower frequencies can be studied with a far better resolution. This study combines the benefits of cochleagrams with Cepstral analysis. This is because, during the generation of a cry, the glottal impulses travel across the vocal tract, which then has a filtering effect on them [48]. The Cepstrum facilitates distinguishing the source and the filter [49], which is desirable for identifying the region of the malfunctioning body organ. GFCCs have also shown promising potential in non-speech classification tasks such as emotion recognition [33]. Regarding the computational costs associated with the extraction of GFCC features, it was shown that by cascading *n* 1st-order Gammatone (GT) filters, the *n*th-order GT filter could be well approximated. In order to attain GFCCs, the cry signal is first windowed into overlapping Hamming filters of 10 ms with 3 ms overlap length, since the performance of the feature extraction step is enhanced, and the non-stationarity of the signal could be neglected in such short frames. Next, in order to pre-emphasize the valuable signal frequencies, the signal passes the GT filters after a fast Fourier Transform (FFT) is applied. The final steps of extracting the GFCCs constitute employing the log function and then the DCT to decorrelate the compressed outputs of the previous steps. For a given frame k, the GFCCs can be computed through Equation (1):

(1) $${\mathrm{GFCC}}_{k}=\sqrt{\frac{2}{N}}\overset{N}{\underset{n=1}{\sum }}\mathrm{GF}\left[k\right]\cos\left(\frac{i\pi }{2N}\;(2c+1)\right)\;1\;\leq \;k\;\leq \;M,\;$$

where $\mathrm{GF}\left[k\right]$ denotes the loudness-compressed response of the Gammatone Filters (GF), and the number of filters is given by N.

*Harmonic Ratio (HR)* has been implemented as a powerful descriptor feature in many applications related to audio classification since it provides high accuracy [50]. The newborn cry has the potential to be studied in terms of its musical aspects in addition to being treated as a pre-speech phenomenon owing to its harmonic components and rhythm and the differences in sound generator organs between newborns and adults [24,51]. By definition, a sound is considered harmonic when a series of frequencies derived from the fundamental frequency as its multiples (called resonance frequencies) are observed in the sound [52]. Several researchers have revealed the presence of harmonics in the cry signals of newborns, and the study carried out by Kheddache et al. [51] précised the harmonic behaviour (the behaviour of resonance frequencies) in pathologic cries, which showed different distributions and patterns among healthy and pathologic cries and among groups of pathologies. More specifically, they concluded that this behaviour depends on the pathology group. Based on these observations, this study evaluates the performance of HR as a potential biomarker for distinguishing between two pathologic groups of cries. HR determines the proportion of the energy of the harmonic segments of the cry signal to the total energy of the cry signal, and four statistical measures of mean, median, interquartile range, and standard deviation were computed based on HR in order to better represent the distribution of this feature across the spectrum of the signal [52]. 

Finally, it is worthwhile to discuss why we chose to fuse HR and GFCC feature sets in this study. We aimed to propose a simple yet effective design that considered both the short-term and spectral behavior of the cry signal. For this purpose, the HR was chosen because it could demonstrate the abnormalities in the cry signal with low computational costs and low feature dimensions, and in addition was shown to demonstrate a meaningful difference between infants diagnosed with RDS compared to other pathology groups. The GFCC feature set was also used as a more robust alternative to the MFCCs that are the most prevalent in the field of audio processing applications. It was shown in [18,19] that the combination of short-term and spectral features provide better classification performance for the study of RDS and sepsis. Furthermore, feature fusion was shown to enhance the performance of the diagnostic system designs for depression data [53] and artifact rejection in neuroimaging data [54] by playing a significant role in enhancing the linear separability through constructing the apropos feature set. Thus, forming a feature vector that merges spectral and short-term and maintains simplicity, robustness, and low dimensionality, is advantageous and interesting to be explored. The feature sets were fused by the means of simple concatenation, and then normalized using a standard normalization. By implementing a fused feature set, we can expect a robust newborn pathology classification performance benefitting from a simpler classification process. Moreover, it would improve the linear separability of various pathology groups within the feature space. The individual feature sets of HR and GFCC were also normalized before being fed into the classifiers.

### 3.4. Machine Learning Classification and Tuning

In this study two classification methods were used, namely SVM and MLP, and both of them were chosen based on their common properties, which are simplicity and cost-effectiveness. The SVM classifier is one of the prevailing algorithms when it comes to the infant cry applications, hence it is often employed as a baseline in many studies to highlight the role of other stages of the design, e.g., how successful the features are and to provide comparability to the classifiers and works of other researchers [15,55,56]. This is because the data in biomedical studies are often very limited and one of the main strengths of the SVM is the ability to efficiently construct complex decision boundaries from limited samples [57]. Moreover, SVM is suitable for a portable and low-cost model design. The MLP classifier has a similar performance to the SVM, the samples are classified by constructing a complex decision boundary. MLP was successfully applied to several studies regarding asphyxia, which also involves the respiratory system [58,59,60]. Hence, it would be beneficial to investigate MLP in the diagnosis of RDS as well. Moreover, MLP is amongst the simplest NN classifiers. The application of MLP is lucrative to assessing the potential of more advanced NNs with more data in the future.

#### 3.4.1. Support Vector Machine (SVM)

SVMs are among the most recognized classification methods implemented for the study of audio signals. Both linear and nonlinear classifications can be performed via SVMs, which are categorized as high precision supervised learning algorithms. The classification procedure of SVM consists of constructing a hyperplane that forms the farthest distance between the data points of different classes. For the case where the data points are not linearly separable, kernel functions are implemented. In this study, a Radial Basis Function (RBF) kernel was chosen, which presumes the neighboring points belong to a similar group and calculates the Euclidean distance between two given points in the feature space [15].

#### 3.4.2. Multilayer Perceptron (MLP)

**Figure 2:**
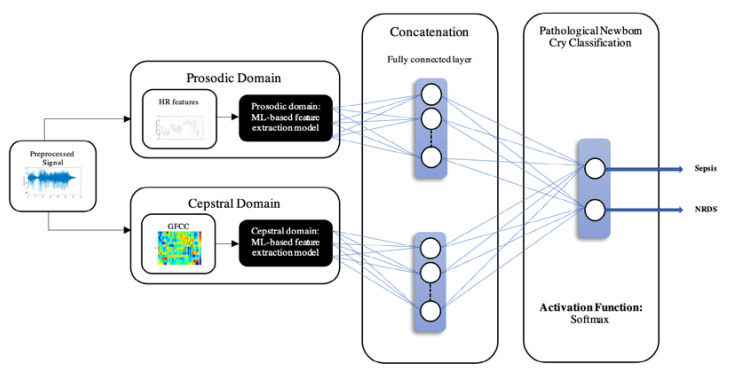
Block diagram for NCDS with MLP classifier.

The general algorithm of a MLP consists of four steps: feeding the pattern to the network, feeding forward across the following layers, updating weights through a backpropagation method, and finally optimizing using an optimization function [61]. MLP constructs a linear decision boundary for classification, and similar to SVM, a hyperplane is constructed so that the decision boundary has the minimum distance from misclassified points [62]. The Root Mean Square Propagation (RMSprop) was used as the optimization function that helps minimize this distance by tuning the backpropagation weights [63]. In order to evaluate the feasibility of employing neural networks for discriminating among groups of pathologies, a 7-layer MLP classifier was designed and proposed. Figure 2 shows the system design with the MLP classifier. With the use of HPO methods, the MLP was configured and tuned for each experiment. The input layer had the same number of neurons as the input feature vector (4, 13, and 17 neurons for HR, GFCC, and fused feature sets, respectively). Next, a 128 node fully connected layer was followed by a normalization layer and a hyperbolic tangent activation function. The activation function decided whether the neuron would fire. Next, another fully connected layer consisting of two nodes that corresponded to the number of output classes (Septic vs. RDS) was included. Finally, a sigmoid layer was used to convert the raw outputs of the previous layers into meaningful class probabilities between the range of [0, 1], and these probabilities were then fed to the classification layer where the decided label was produced. Training iterated with a learning rate of 0.001 through 120 epochs, and then validated by 15% of all the data, with 30% of the data randomly split for testing, and 55% of the data used for training. 

#### 3.4.3. Hyper-Parameter Fine-Tuning and Evaluation Measures

Attaining desirable classification performance, as well as low error rates, is the goal and the main challenge of all classification problems; hence, the fine-tuning methods of HPs were introduced to serve this purpose.

Each experiment requires its own HP tuning since the feature matrix dimensions vary so that the classifier is tailored to fit the task. Furthermore, the HP fine-tuning methods replace human interference in determining classifier HP configuration, which includes random search, grid search, and Bayesian HPO approaches. In this study, the grid search method was used to fine-tune the classifiers’ HPs, where it selected an optimum value for HPs from a limited set [64]. The HPs selected for SVM fine-tuning were the γ and C, whereas the initial learn rate, L2 regularization, and the number of epochs were tuned for MLP. 

In order to assess the ability of the proposed design in discriminating between the two groups of pathologies, several evaluation measures should be considered. Generally, the accuracy measure is the most prevalent measure in all systems, which is equal to the ratio of correct predictions to all the observations. The accuracy owes its prevalence to simplicity in calculation and understanding, but it is not informative in terms of class assessment and missed cases; therefore, other measures were introduced and studied. Table 2 presents a number of these measures used in this study [19]. 

## 4. Results and Discussion

This study targets the distinction between two entangled groups of pathologies in newborns for the first time in NCDS designs to the best of our knowledge. The aim of this study was to develop an early alert for the detection of sepsis and RDS, which are among the top newborn mortality causes around the world. Assessing the potential of analyzing acoustic features of the cry signal as a biomarker, through simple and accessible tools, was the priority of the proposed NCDS. Our dataset was recorded through a handheld recorder in the presence of noise with no prespecified conditions in maternity rooms and NICUs. Furthermore, newborns from different races, origins, genders, and various reasons of crying participated in our study which makes it comprehensive. Moreover, this study combined features that were conventional in musical applications of HR with the biologically inspired features used in speech-processing applications, and GFCCs that belonged to two levels of short-term and spectral. Additionally, with the help of HP fine-tuning, the classifiers were tailored to fit each of the presented experiments.

Various audio recognition, speech, and music processing systems benefit from sophisticated and complex deep-learning models, whereas in biomedical applications, the use of these designs depend on data availability. Data acquisition and collection are among the most significant challenges in biomedical research; when it comes to observing certain pathological groups, the probability is not deterministic in any given period of time. There is no way of knowing whether the newborns admitted to a hospital on a certain date would be diagnosed with the pathology groups subject to research. Nevertheless, obtaining the ethical and technical requirements to include data from any participant adds to the challenge of data acquisition. Therefore, this study benefits from SVM as a desirable and successful approach in NCDS designs and explores the use of a MLP neural network in order to assess the further potential for using other NN models in future works.

As mentioned in previous sections, the NCDS was designed and analyzed with the EXP dataset. The MLP and SVM classification approaches were used to identify septic newborns from RDS, and the feature sets were employed individually and also after their fusion. In order to fuse the features a simple concatenation followed by standard normalization was performed, so that the performance of the feature set implementing both modalities (short-term and spectral) would be compared to the individual feature sets. Furthermore, the classifiers were fine-tuned using the grid search hyperparameter (HP) optimization. In this case, γ and C were tuned for the SVM classifier, while the HPs of L2 regularization, initial learn rate, and number of Epochs were optimized for the MLP. In order to fully investigate the potential of HP fine-tuning, the range for each HP was determined for the optimization process, Table 3. Elaborating the reasons behind choosing which HPs were tuned in this study would be of essence.

*Initial Learning Rate* is the most significant HP to tune in neural networks. Following each iteration of estimating the error yielded after updating the weights, the learning rate determines how much of an adjustment the model requires. 

Selecting the optimal learning rate is a trade-off between computational time and finding the optimal solution. Larger learning rates lead to the faster convergence of the model to the suboptimal solution, whereas a small learning rate calls for a higher number of epochs. Therefore, we should tune the number of epochs as well [65]. 

*Number of Epochs* determines the number of changes in the weights of the network; increasing and decreasing the number of epochs may lead to the underfitting and overfitting of the model. Therefore, while tuning other HPs of the network, it is important to select the optimal number of epochs correspondingly. The optimal selection of the number of the epochs allows for the termination of the training process before the elevation of the validation error [66]. 

*L2 Regularization*: In order to prevent machine learning techniques from encountering overfitting, regularization methods were introduced [67] so that by adding a penalty factor to the large weights, the complexity of the overall design was reduced. L2 regularization is amongst the most prevalent methods of regularization. The value of regularization HP should be selected in such a way that both overfitting (associated with small regularization value) and underfitting (associated with large regularization value) are prevented [68]. 

As for the SVM classifier, both γ and C should be tuned. A higher value of the C would prioritize decreasing the support vectors count due to the fact that they each add to the optimization costs, while lower values of C lead to a higher support vector count and thus, larger margins. The γ HP determines the simplicity of a SVM model; higher values correspond to a curvier decision plane, which closely follows the data, whereas a small γ means a simpler model with flatter decision plane. γ in fact signifies the speed of lowering the domination of each point as the distance grows [69].

We conducted three experiments to evaluate the system performance, the role of fused features, and the role of each feature set. Table 4, Table 5 and Table 6 present the results of the evaluation of the proposed design based on these experiments.

The results for the evaluation of the HR feature set are presented in Table 4. The HR feature set proved to be a successful feature in the analysis of the cry signal, since with only 4 elements, the NCDS could yield a 71.03% accuracy. However, the MLP classifier did not converge for the HR feature set. This result was unsurprising since this feature set has a low dimensionality of only 4 elements. Therefore, increasing the number of features could solve this challenge, as presented in Table 6. 

Moreover, this feature set could also obtain fair performance in terms of recall and precision. The recall measure is of great significance in exploring the pathologies, since it demonstrates the share of true septic (or RDS) cases among all the samples. Precision shows the probability that NCDS will predict a septic (or RDS) case correctly. These two measures owe their importance to the fact that true diagnosis and timely treatment of the pathology have a considerable effect on the survival chances of the newborn. 

The GFCC feature set remarkably attained a high performance as an individual feature set with both classification methods, Table 5.

**Figure 3:**
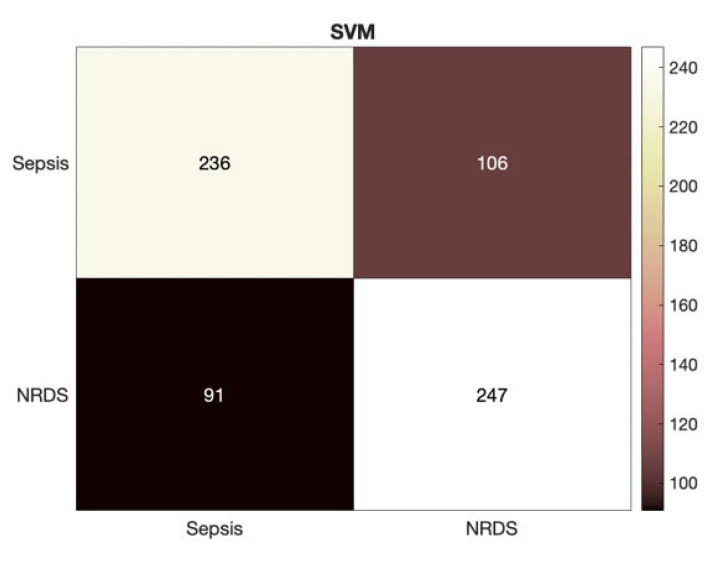
Heatmap for the SVM classifier using the HR feature set.

Increasing the number of features resulted in the convergence of the MLP classifier as expected; however, the SVM outperformed MLP across all evaluation measures. It can also be seen that the performance of the NCDS with GFCC feature set was superior to the HR feature set by more than 10% in accuracy. Figure 3 depicts a more detailed look at the results of identifying the septic and RDS cases via HR feature set through presenting the heatmap for the SVM classifier.

In the final experiment, we fused the previous features to assess the performance of NCDS in discriminating between RDS and septic newborns, Table 6. The addition of the HR features resulted in an enhancement of more than 2% across all the evaluation measures for both classifiers compared to the GFCC feature set. These results are promising due to two main points: (1) improving the performance where the results are already at more than 90% would be difficult, and our design gained more than 2% enhancement. (2) this enhancement is consistent across all the evaluation measures investigated. Similar to the GFCC feature set, the SVM transcended the MLP throughout the evaluation measures. 

**Figure 4:**
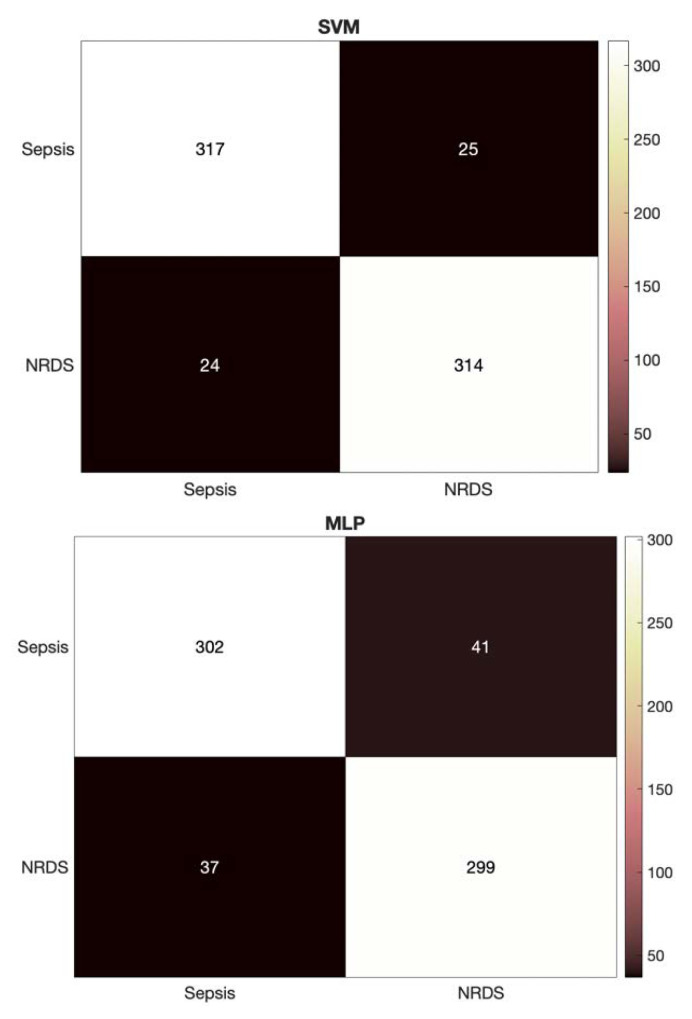
Heatmaps for the SVM and MLP classifiers using the GFCC feature set.

**Figure 5:**
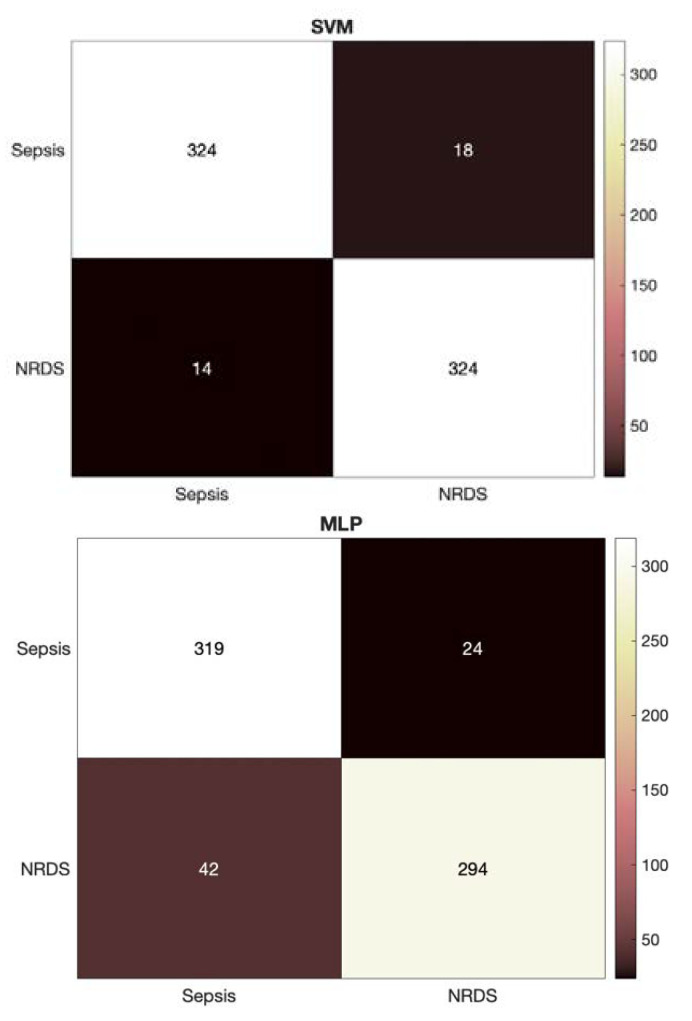
Heatmaps for the SVM and MLP classifiers using the combined feature set.

Similar to the HR feature set, the detailed heatmaps for the GFCC and combined feature sets using each of the classifiers are presented in Figure 4 and Figure 5, respectively. These heatmaps show how the data are distributed across the classes and provide a deeper look into the predictions made by the NCDS. 

**Figure 6:**
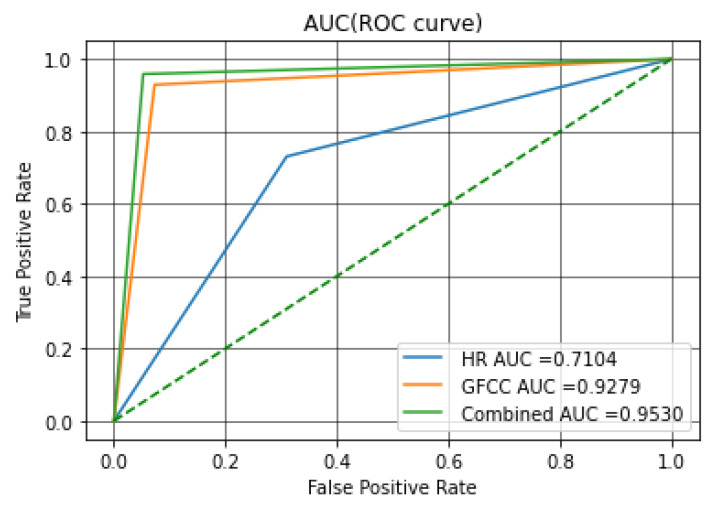
AUC-ROC for the SVM classifier using each feature set.

Finally, comparison of the Area Under Curve (AUC) of the Receiver Operator Characteristic (ROC) for the experiments in this study would help further assess the performance of different architectures. Figure 6 shows the ROC curves for the SVM classifier. The ROC curve shows the true positive rate (TPR) on the vertical axis and the false positive rate (FPR) on the horizontal axis. FPR is also an important measure, since it represents the probability of a false alert. The area under curve (AUCs) of ROCs is an indicator of model performance which will be discussed later in this section.

As can be seen through all evaluation measures, the fused feature set achieved the highest results with both classifiers. The study of the AUC is salient in terms of statistical analysis, since it demonstrates the probability of ranking any positive sample is higher than any negative sample, the same as Wilcoxon test of ranks [70] in order to compare the classifiers; the ROC curves are summarized in a single scalar, the AUC. The AUC is always between 0 and 1 since it is defined as a share of the area of the unit square [71]. Any practical and acceptable classifier should have an AUC of more than 0.5 since the random guessing is equal to the diagonal line in the ROC curve that crosses (0, 0) and (1, 1); the closer values of AUC to 1 translate to better performance of the classifier. In other words, the AUC signifies the ability of the system in distinguishing between the two classes which is the main goal of this study [72]. 

Two main goals were introduced for this study: (1) finding the optimal feature set and study the effect of combining spectral and cepstral features. (2) finding the best classification algorithm that fits our problem/challenge.

**Figure 7:**
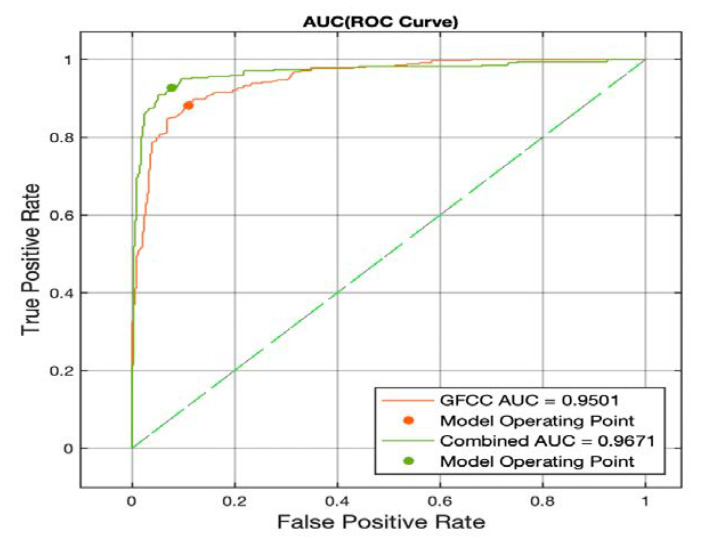
AUC-ROC for the MLP classifier using the GFCC and combined feature sets.

Through comparing the AUCs resulting from analyzing the introduced feature sets in this study with the SVM classifier, the role of feature fusion in studying the pathologic infant cries became clear. It is shown that implementation and combination of different modalities can enhance the performance of the system, thus achieving the first goal. Concerning the second goal, it was shown that the MLP classifier was outperformed in terms of evaluation measures for all feature sets; therefore, as a final discussion point, we compared the AUC-ROC of the best feature sets of the SVM and MLP classifiers. Figure 7 illustrates the ROC curve for the MLP classifier; as can be seen from Figure 6 and Figure 7, the MLP showed better performance in terms of the AUC measure. This is an interesting result since it suggests two points: (1) the study of the ROC curve is essential for analyzing the binary classification problems since the evaluation measures might not describe all the aspects. (2) the MLP classifier shows great potential in studying the pathological infant cry signals since it has better performance in the separation of the two classes and should be considered for future studies. Finally, it can be seen that the superiority of the combined feature set is consistent across both classifiers as the MLP classifier also has a 0.17 increase in the AUC by implementing a combination of the features.

There are few studies analyzing newborn cry signals to diagnose sepsis. Recently, two groups of researchers studied sepsis based on processing the newborn cry signals; however, they both focus on detecting septic newborns from the healthy group, whereas this study aims to target distinguishing between two pathological groups for the first time. The study presented by Matikolaie et al. [18] investigated the role of prosodical characterization of the cry signal in detecting sepsis which accomplished 86% as their best F-score. Furthermore, Khalilzad et al. [19] explored the potential of a NCDS in diagnosing sepsis by incorporating entropy-based features and fuzzy entropy feature selection, which attained 89.70% as their best F-score for the expiration cry segments. We believed that with sepsis being one of the globally leading post-partum mortality causes, there is a need for more in-depth studies that probe other perspectives of this pathology. Hence, this study could be complementary to the previous studies to give another means and modality of studying sepsis by comparing it to another cognate pathology. 

The respiratory distress syndrome (RDS) suffers from a similar research gap; the existing literature on processing RDS cries is scarce. There are few studies target studying RDS as a single pathology group; Matikolaie et al. [20] proposed a NCDS to detect newborns suffering from RDS from the healthy and obtained 73.80% accuracy. Chittora et al. [73] presented a spectrographic comparison of the RDS cries, where a double harmonic break was presented, suggesting that resonant study of the cry signal would be helpful in analyzing the RDS cries. Moreover, Lederman et al. [74] classified the preterm infants suffering from RDS from healthy preterm infants and achieved a 63% accuracy using hidden Markov models. Finally, Alaie et al. [11] obtained 69.59% accuracy by GMMs using the boosting mixture learning method for the detection of infants diagnosed with RDS; in another experiment, they formed a subset of pathological newborns suffering from multiple pathologies such as RDS, heart problems, blood abnormality and neurological disorders as a single pathological group to be detected from healthy newborns and gained an accuracy of 85.21%. As mentioned above, all discussed research focused on the identification of RDS/Sepsis from healthy; however, to the best of our knowledge there is no prior work on distinguishing between two (or more) pathology groups. Nevertheless, despite the entangled nature of the two pathologies studied here, our design was able to outperform all of the previous studied on sepsis and RDS cry signals by achieving 95.3% for accuracy. Similar to any other study in this field, this study also faced multiple challenges. Although we attempted to study the cry signals regardless of race, origin, and other factors such as cry stimuli, the designed NCDS has room to be further developed with more data. Furthermore, employing explainable AI, such as LIME, might help to better analyze the contribution of different features to the final result; thus, it will be considered in our future works. 

This study had several achievements; it provided a proof for the concept of distinguishing between different pathology groups based on only cry signals, as well as further highlighting the benefit of combining features from different levels. Furthermore, by using proper feature manipulation, normalization, and HP fine-tuning, our machine learning design was able to achieve results similar to the more complex and resource expensive methods in the literature by attaining an accuracy and F-score of up to 95%. The high values of recall demonstrate the success of our design in detection of the true pathology group. 

## 5. Conclusions and Future Work

This paper aimed to investigate RDS and sepsis as two of the pathologies associated with high mortality rates of neonates across the world through machine learning-based methods. These two pathology groups require in-depth and extensive clinical tests to be diagnosed, which calls for the development of a non-invasive tool such as the one suggested in this study. The novelty of the proposed design lies in removing the need for any extreme data collection or analysis tools by employing a commercial handheld recorder for data acquisition with no well-defined conditions, as well as using conventional machine learning techniques and combining them in such a way that the performance of the system is comparable to the highly complex and recent methods. This study proposed an early alert for detecting and discriminating two entangled groups of pathologies for the caregivers of the newborn and the medical staff in deprived areas of the world suffering from high newborn mortality rates. 

The classifiers in this study were tuned for each experiment and all the feature sets were normalized before being fed to the classifiers. The cry signals were studied from a musical perspective through the HR feature set and from a speech processing aspect by means of the GFCC feature sets. Moreover, these features were from two different levels that also investigated the short-term and the spectral behaviour of the cries. The combination of these two feature sets improved the overall performance of the system, and the final accuracy and F-score were as high as 95%.

In this research work, we have noticed that training deep learning approaches requires a large size of diverse samples of infant pathologies. Therefore, increasing the number of samples is desirable for introducing deep learning models. Instead of using GFCC features modality only, we have also seen that combining HR features and GFCC features has positively contributed to improving the classification rates by 2.35% and 2.21% using SVM and MLP, respectively. Nonetheless, integrating other features from other domains that improve the linear separation ability will be further investigated in our future works. As mentioned before, it has been shown that extracting spectrogram features includes important information or characteristics in classifying infant crying signals [34,35,36,37]. Combining spectrogram features along with the prosodic and cepstral features will be one of our future works. Whether the features will be fused prior to training or within the learning process is also an open question. 

Our next work will be based on proposing a multimodal fused model for the diagnosis of different infant pathologies leading to an accurate NCDS. This will include increasing the dataset by introducing new pathology types, extracting more robust features from different domains, fusing them with appropriate ratios, and then generating a new combined feature set that improves the discrimination ability. The feature analysis will be based on more sophisticated techniques, such as deep learning approaches. Therefore, studying and finding novel deep learning architectures, such as CNN and DFNN, with the use of combined features will also be considered.

## Figures and Tables

**Table 1:** The dataset description.

	Septic	RDS
**Gender**	11 Males and 6 Females	10 Females and 23 Males
**Average sample length**	71 milliseconds	74 milliseconds
**Babies Ages**	1 to 53 days old	
**Prematurity**	Term	
**Gestational age**	38 ± 1 week	
**Number of samples**	2264 (1132 each)	
**Origin**	Canada, Haiti, Portugal, Syria, Lebanon, Algeria, Palestine, Bangladesh, Turkey	
**Race**	Caucasian, Arabic, Asian, Latino, African, Native Hawaiian, Quebec	
**Reason of crying**	Birth cry, hunger, dirty diaper, discomfort, needs to sleep, cold, pain	

**Table 2:** The evaluation measures and their formula.

*Evaluation Measure*	*Formula*
***Accuracy***	$\frac{\mathrm{TP}+\mathrm{TN}}{\mathrm{TP}+\mathrm{FP}+\mathrm{FN}+\mathrm{TN}}\times 100$
***Sensitivity***	$\frac{\mathrm{TP}\;}{\mathrm{TP}+\mathrm{FN}\;}\times 100$
***Precision***	$\frac{\mathrm{TP}\;}{\mathrm{TP}+\mathrm{FP}\;}\times 100$
***F-score***	$\frac{2\mathrm{TP}\;}{2\mathrm{TP}+\mathrm{FP}+\mathrm{FN}\;}\times 100$

**Table 3:** The pre-defined ranges for HP fine-tuning.

Classifier	Parameter	Selected Range	Value Type
MLP	Initial learning rate	[0.0001, 1]	Logarithmic
	L2 Regularization	[0.0001, 0.001]	Continuous
	Number of Epochs	[50, 200]	Integer
SVM	γ	[0.1, 0.25, 0.26, 0.3, 0.5]	Categorical
	C	[0.5, 1, 2, 4, 5]	Categorical

**Table 4:** The results for the evaluation of the HR feature set.

Feature Set	Classifier	Accuracy	Precision	Recall	F1-Score
HR	SVM	71.03%	0.71	0.71	0.71
	MLP	N/A	N/A	N/A	N/A

**Table 5:** The results for the evaluation of the GFCC feature set.

Feature Set	Classifier	Accuracy	Precision	Recall	F1-Score
GFCC	SVM	92.94%	0.93	0.93	0.93
	MLP	88.51%	0.88	0.89	0.89

**Table 6:** The results for the evaluation of the combined feature set.

Feature Set	Classifier	Accuracy	Precision	Recall	F1-Score
GFCC + HR	SVM	**95.29%**	**0.95**	**0.95**	**0.95**
	MLP	92.49%	0.92	0.92	0.92

## Data Availability

Not applicable.
